# An Ultrasound Investigation of Tongue Dorsum Raising in Children with Cleft Palate +/- Cleft Lip

**DOI:** 10.1177/10556656231158965

**Published:** 2023-02-27

**Authors:** Joanne Cleland, Marie Dokovova, Lisa Crampin, Linsay Campbell

**Affiliations:** 1 150865 School of Psychological Sciences and Health, University of Strathclyde Glasgow, Glasgow G1 1XQ, UK; 2 Speech and Language Therapy, Children's Hospital, Glasgow, G51 4TF, UK

**Keywords:** articulation, Pediatrics, Speech disorders, Tongue, Ultrasound

## Abstract

**Objective:**

This study aimed to determine whether increased raising of the back of the tongue is evident in children with repaired cleft palate with or without cleft lip (CP+/-CL). We hypothesized that children with CP+/-CL would show increased raising of the tongue dorsum, a compensatory pattern.

**Method:**

Secondary data analysis of mid-sagittal ultrasound tongue imaging data from 31 children with CP+/-CL and 29 typically developing children were used. We annotated the consonants /ʃ, t, s, k/ at the point of maximum constriction in an /aCa/ environment. Children with CP+/-CL said the tokens 10 times, typically developing children said them once. We automatically fitted splines to the tongue contour and extracted the Dorsum Excursion Index (DEI) for each consonant. This metric measures the relative use of the tongue dorsum, with more posterior consonants having higher values. We compared DEI values across groups and consonants using a linear mixed effects model. DEI was predicted by the interaction of consonant (baseline: /ʃ/) and speaker type (baseline: TD), including by-speaker random slopes for consonant and random intercepts for speaker.

**Results:**

Overall DEI was not higher in children with CP+/-CL compared to typically developing children. Between groups the only significant difference was the position of /k/ relative to /ʃ/, where the difference between these two consonants was smaller in the children with CP+/-CL.

**Conclusions:**

There was no support for the hypothesis that increased raising of the tongue dorsum is a common characteristic in children with repaired CP+/-CL. However, individual children may present with this pattern.

## Introduction

Speech differences are common in children with cleft palate +/- lip (CP+/-CL) and can persist after surgery.^1^ Even when velopharyngeal function is adequate, lingual articulation may be affected due to the shape of the maxillary arch; compensatory strategies (such as blocking a fistula, or compensating for velopharyngeal dysfunction); and related articulatory mislearnings.^2^ As part of these errors, speakers with CP+/-CL may show increased raising of the back or body of the tongue^3^ compared to speakers without CP+/-L.

This pattern of maintaining a high tongue body position and articulating more with the back of the tongue can lead to backing errors, for example producing anterior consonants such as /t,d,n/ at a more posterior place of articulation. Alternatively, speakers may produce double articulations where both the tip and the body of the tongue articulate simultaneously.^4^ It is also suggested that the phenomenon of maintaining a high tongue body position, even when anterior consonants are produced at the correct place of articulation, could also arise from an attempt to compensate for velopharyngeal dysfunction. Trost^3^ terms this “lingual assistance”. This high tongue body posture may persist after successful surgery to improve velopharyngeal closure due to early mislearning.^5^ This can lead to difficulties with intelligibility and potentially a tongue position that is different from speakers without CP+/-CL.

The standard method for identifying most compensatory articulations, including backing errors, in CP+/-CL is with perceptual evaluation.^1^ Using this method, the Speech and Language Therapist (SLT) listens to the speaker and uses symbols from the International Phonetic Alphabet, and the extended version for disordered speech, to transcribe and then categorise errors. In many cases the errors may be straightforward substitutions with phonemes of the target language, however, in the case of CP+/-CL, the errors are often sounds which do not occur in the target language. For example, backing to a pharyngeal place of articulation is common, as are glottal articulations and these speech sounds are not phonemes of English, making them more difficult to detect and transcribe in English-speaking children.^3^ Nevertheless, narrow phonetic transcription can be reliably used to identify backing errors,^1^ even if there is some disagreement on the particular symbol used. However, in the case of an abnormally high or retracted tongue position during anterior consonants, this is less likely to be transcribed as an incorrect consonant production but could be perceived as a problem with oral resonance or voice quality.^6^ While abnormal resonance can be detected perceptually, attributing any difficulties in this area to tongue posture is difficult without instrumental analysis. Likewise, acoustic analysis can be used to identify errors such as pharyngeal fricatives^7^ but there is no easy way to use it to identify increased raising of the tongue body. This can be achieved with articulatory instrumentation such as x-ray imaging.^8^ Michi and colleagues^8^ demonstrated that the tongue back was raised towards the velar region of the hard palate and the anterior soft palate in children with CP+/-CL who also presented with increased contact or retracted tongue-palate contact patterns measured with electropalatography (EPG). However, EPG only allows visualization of tongue palate contact from the alveolar to velar region. It does not allow visualization of articulations where the tongue does not make full contact with the hard palate, such as open vowels, or post-velar articulations, such as pharyngeals which are common in CP+/-CL. Nor does it allow visualization of a raised tongue dorsum, unless this results in tongue palate contact. Gibbon, Smeaton-Ewins and Crampin^6^ demonstrated that children with CP+/-CL are more likely than typically developing children to produce vowels, particularly high vowels, with abnormal amounts of tongue palate contact. Therefore, increased tongue palate contact may be a key indicator of overuse of the tongue dorsum and has been reported extensively in the literature.^4^

However, there are limitations with using EPG, and indeed x-ray which uses ionizing radiation, to identify increased raising of the tongue dorsum. Firstly, EPG requires a custom-made dental plate for each user. Secondly, this dental plate may fit for only a limited length of time in children who are under-going orthodontic treatments or changing dentition.^9^ Both of these criteria may have led to selection bias in previous studies of articulation in CP+/-CL, that is children were included in studies where they were candidates for EPG therapy, in other words children with more severe or intractable speech problems.^9^ Ultrasound tongue imaging (UTI), however, requires no individualized hardware, making it easier to apply to larger groups of speakers with CP+/-CL. UTI can be used to image the tongue in either a mid-sagittal or coronal view. Moreover, unlike EPG, it allows visualization of tongue shape and movement, with articulations such as pharyngeals clearly visible. The mid-sagittal view is most used for both research/assessment^10^ and biofeedback.^11^ In this view most of the tongue is visible, including the root. However, the tongue tip and part of the root are in shadow from the mandible and hyoid bone respectively. Ultrasound videos of articulations can be viewed at seeingspeech.ac.uk.^12^ UTI has suffered from slow adoption into the CP+/-CL clinic for a few reasons. Firstly, analyzing ultrasound is difficult and time consuming.^10^ In order to analyze images, the surface of the tongue must be tracked, and then co-ordinates exported and analyzed. Until recently this has been a time-consuming process, with annotators drawing lines on the images by hand. This problem is now largely solved with automatic tracking available,^13^ in some cases, including estimation of the tip of the tongue's position.^14^ A small number of studies have used UTI for measuring articulation in speakers with CP+/-CL.

Bressmann and colleagues^15^ investigated compensatory articulations during productions of /k/ in five speakers with CP+/-CL. By visually inspecting the ultrasound images, they identified compensatory articulations including pharyngeal stops and mid-palatal stops. A larger study of 35 children with CP+/-CL took a similar visual inspection approach. Cleland et al.^16^ used UTI as an additional modality to support the phonetic transcription of speech from individuals with CP+/-CL. They showed that using UTI increases the reliability of phonetic transcription and allows the SLT to identify compensatory or disordered articulations such as increased contact; retraction; fronting; complete closure; uvular or pharyngeal articulations; double articulations; increased variability; abnormal timing; and retroflexion. However, neither of these studies attempted to quantify lingual articulations, which may be important for comparisons to typically developing speakers. A further small study of two children with submucous CP+/-CL used articulatory t-tests to compare perceptually neutralized phones and found some evidence of covert contrast.^17^ However, this study focuses on within-speaker comparisons, rather than comparisons to typical speakers. In fact, instrumental articulatory studies of speech from people with CP+/-CL have been characterized by not only a lack of normative data, but also by a lack of comparisons between speakers with overt speech disorders and those with no obvious speech difficulties.^4^ In the case of EPG, this has been because the cost of EPG may lead clinicians, and to a lesser extent researchers, to prioritise EPG for speakers where it can also be used as a biofeedback therapy. These issues are overcome with UTI which can be used with multiple speakers with no additional costs.

For UTI to be useful for measuring raising of the back of the tongue, metrics are required which allow comparison between speakers and between speech materials. Zharkova^18^ suggests a range of metrics for quantifying tongue shape and movement in speakers with CP+/-CL, although this particular study, and a follow-up,^19^ do not test these with speakers with CP+/-CL. Of these the “Dorsum Excursion Index” is most relevant for measuring raising of the tongue dorsum.

### The Dorsum Excursion Index

**Figure 1.**
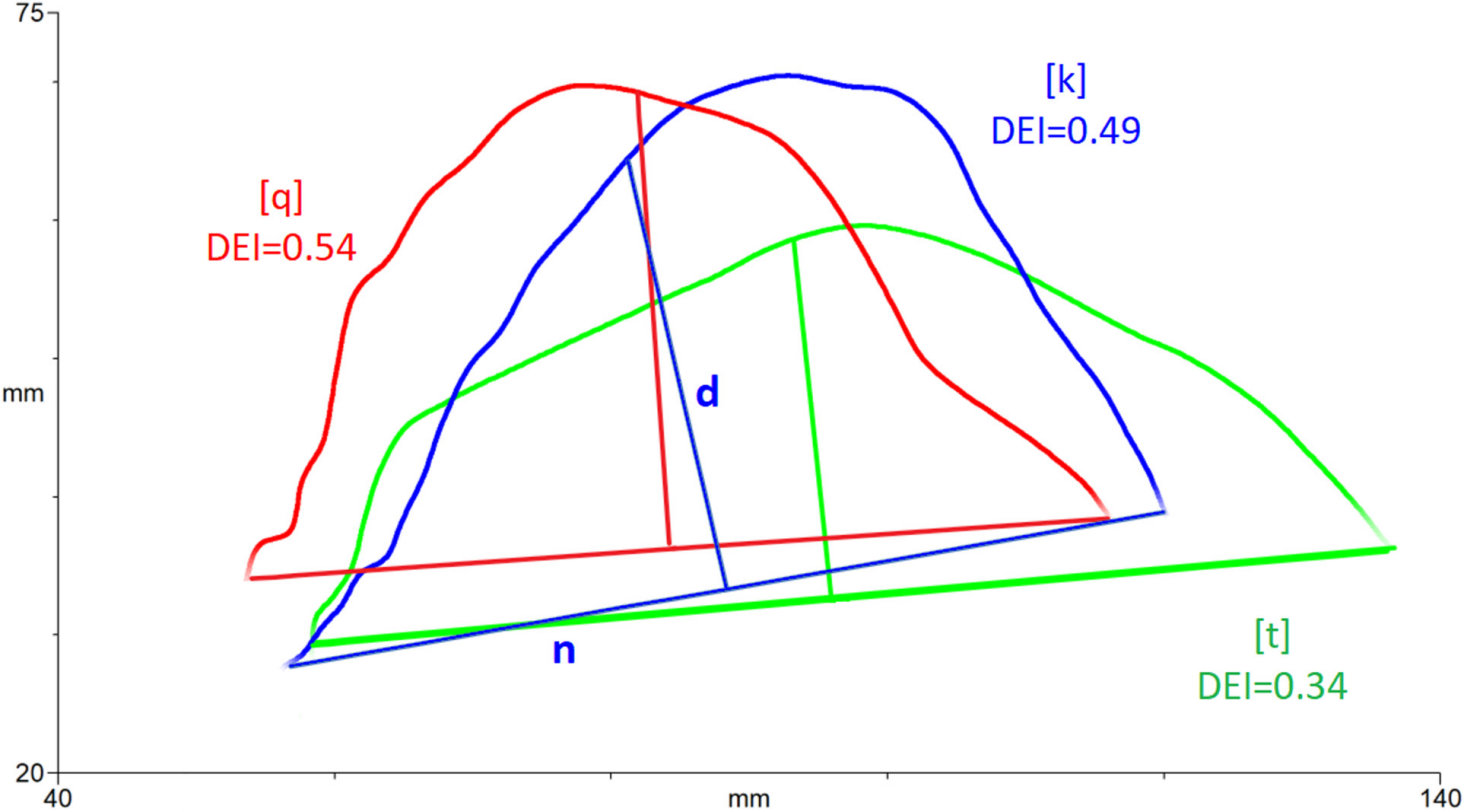
Example tongue splines for calculation of dorsum excursion index. Blue = /k/, Green = /t/, red = [q], with the tongue tip to the right. A line, n, is drawn between the ends of the spline and the midpoint found. A perpendicular line, d, is drawn and the dorsum excursion index is calculated as d/n. DEI values are green = 0.34, blue = 0.49 and red = 0.54.

The Dorsum Excursion Index (DEI) is a scalar metric and therefore not sensitive to differences in rotation, translation, or tongue size,^10^ making it a useful measure for comparing children of different sizes and ages. The measure is designed to directly assess the extent of tongue dorsum, or back, involvement during articulations.^18^ For the purposes of the measure, Zharkova (2013:485) defines the location of the tongue dorsum as “opposite the midpoint of the straight line traced between two ends of the midsagittal tongue curve” (see figure 1).

To calculate the DEI, first, the surface of the tongue at the point of interest- either mid-consonant or vowel or the frame of maximum excursion- is traced with a “spline” and then a straight line (n) is drawn between each end of the curve and the midpoint found. A perpendicular line (d) is drawn from this midpoint to the spline and a ratio (d/n) calculated. Figure one illustrates calculation of DEI for a /t/ articulation (green), a /k/ articulation (blue) and a [q] articulation (red) produced by a typical adult speaker where DEI is the ratio of d/n. Since DEI is a ratio measure which can be calculated irrespective of translation or rotation, it is important to note that it is a measure of tongue shape, not place. That is, more bunched shapes have higher DEI values and flatter shapes have lower DEI values, but retraction or place of articulation cannot be assumed.

A follow-up paper^19^ to Zharkova^18^ validates the DEI scores with six typical adult speakers. In these speakers, DEI for /k/ is greater than other consonants, particularly within the context of an /a/ vowel, although the measure did not so readily differentiate other consonants from each other. A higher DEI value would correspond to sounds that are produced both high and with the back of the tongue, while sounds produced in other positions within the oral cavity (front, or in particular back and low) would yield lower DEI values. Given that the DEI was designed specifically to measure increased raising of the tongue dorsum in speakers with CP+/-CL,^18^ but has not yet been tested with this population, the current study uses this metric to compare speakers with CP+/-CL to typically developing speakers.

### Ethics

Ethical approval for this study was granted by the first author's university and by the NHS West of Scotland Research Ethics Committee. All participants and their carers gave signed consent.

### Aims and Hypotheses

This study aims to determine whether increased raising of the tongue dorsum is evident in children with repaired CP+/-CL, as reported in previous studies.^4^ We aim to compare children with CP+/-CL to children without CP+/-CL, by measuring DEI from ultrasound tongue images in a range of lingual high-pressure consonants. We also aim to report DEI values for typically developing children to supplement the data currently available for six typical adult speakers^19^ and provide norms for future studies.

The hypotheses are follows:

1. DEI will be significantly higher across all consonants in children with CP+/-CL, representing increased raising of the tongue dorsum
2. DEI will be higher in both groups for consonants involving the tongue dorsum, specifically /k/.

## Materials and Methods

This study makes use of two open access data sets from the Ultrasuite corpus^20^: the Ultrax Typically Developing and the Cleft data set. Both comprise high-speed UTI recordings of speech materials designed to sample consonants of English for speech assessment purposes. Since we make use of pre-existing data, there were differences in the speech materials and recording set up for both groups of speakers which we describe below.

### Speakers with Cleft Palate +/- Cleft Lip

This dataset comprises recordings from 39 children with CP+/-CL aged three to 12 (M = 7;0, 2;4). These data are also reported in Cleland et al.^16^ Data were collected from children attending routine appointments over a 12-month period at a specialist cleft speech and language service. Inclusion criteria were syndromic or non-syndromic CP+/-CL, age 3–15 years, and English spoken at home and/or in school. The dataset includes children with all levels of intelligibility, including children with only mild speech problems. Children with cleft lip only, severe learning disability, or no speech were excluded from the dataset. Of the 39 participants, four participants’ datasets were excluded due to issues of audio and ultrasound video synchronisation, three participants’ datasets did not include sufficient ultrasound video for analysis, one participant's ultrasound was of insufficient quality and one participant's dataset was corrupted. This left 31 participants for analysis. Table 1 contains biographical and medical information for the speakers with CP+/-CL.

**Table 1.** Demographic data for children with Cleft Lip and Palate and typically developing children.

Dataset	Participant No.	Sex	Age (years; months)	Cleft type	Additional medical diagnoses	Languages
Cleft	01	M	10;05	BCLP	none	English
Cleft	03	F	05;01	UCLP	none	English
Cleft	05	M	10;03	UCLP	none	English
Cleft	06	M	09;08	UCLP	none	English
Cleft	07	M	09;02	UCLP	none	English, Spanish
Cleft	08	M	04;07	UCLP	none	English
Cleft	09	M	09;08	UCLP	cluttering/stuttering	English
Cleft	10	M	07;07	UCLP	none	English
Cleft	11	M	04;05	CP	none	English
Cleft	12	F	05;01	CP	Stickler syndrome	English
Cleft	13	F	05;02	CP	none	Gaelic, English
Cleft	14	F	05;10	BCLP	none	English
Cleft	15	F	04;09	UCLP	tonsil and adenoidectomy	English
Cleft	16	M	09;07	UCLP	none	English
Cleft	17	F	04;04	BCLP	none	English
Cleft	18	F	05;11	UCLP	none	English
Cleft	19	F	03;09	CP	Treacher Collins syndrome	English
Cleft	20	F	07;05	CP	none	English
Cleft	21	M	09;11	CP	none	English
Cleft	22	M	10;01	CP	none	English
Cleft	23	F	12;02	BCLP	none	English
Cleft	24	M	06;05	CP	Stickler syndrome, micrognathia	English
Cleft	25	M	04;11	CP	none	English
Cleft	26	M	04;04	BCLP	none	English, Turkish
Cleft	27	M	04;00	UCLP	none	Azerbaijani, Persian, English
Cleft	28	F	08;09	BCLP	none	English
Cleft	29	F	07;09	CP	Pierre Robin, micrognathia	English
Cleft	30	F	07;07	CP	none	English
Cleft	31	F	05;04	CP	none	English
Cleft	32	M	05;08	UCLP	none	English
Cleft	33	M	06;04	UCLP	developmental delay	English
Cleft	34	M	05;03	CP	Pierre Robin	English
Cleft	35	M	03;07	UCLP	none	English
Cleft	36	M	05;10	BCLP	none	English
Cleft	37	M	07;00	BCLP	none	English
Cleft	38	F	04;09	CP	none	English
Cleft	39	M	7;00	CP	none	English
Group		22M	M = 7;0			
		15F	SD = 2;4			
Ultrax TD	2	M	11;10	-	none	English
Ultrax TD	3	M	11;09	-	none	English
Ultrax TD	4	F	10;05	-	none	English
Ultrax TD	6	M	9;11	-	none	English
Ultrax TD	7	F	9;10	-	none	English
Ultrax TD	8	F	9;04	-	none	English
Ultrax TD	9	M	8;08	-	none	English
Ultrax TD	10	F	6;09	-	none	English
Ultrax TD	11	F	11;03	-	none	English
Ultrax TD	12	M	8;01	-	none	English
Ultrax TD	13	M	6;08	-	none	English
Ultrax TD	14	F	11;07	-	none	English
Ultrax TD	15	M	12;04	-	none	English
Ultrax TD	16	M	7;11	-	none	English
Ultrax TD	17	F	12;10	-	none	English
Ultrax TD	18	M	10;08	-	none	English
Ultrax TD	19	F	7;02	-	none	English
Ultrax TD	20	M	12;06	-	none	English
Ultrax TD	21	M	11;01	-	none	English
Ultrax TD	22	F	5;08	-	none	English
Ultrax TD	23	M	7;02	-	none	English
Ultrax TD	24	F	12;02	-	none	English
Ultrax TD	25	F	8;07	-	none	English
Ultrax TD	26	M	10;00	-	none	English
Ultrax TD	27	F	7;11	-	none	English
Ultrax TD	29	F	7;03	-	none	English
Ultrax TD	30	F	7;11	-	none	English
Ultrax TD	31	F	10;05	-	none	English
Group		M = 13	M = 9;7			
		F = 15	SD = 2;1			

Table Note: BCLP: Bilateral cleft lip and palate; UCLP: Unilateral Cleft Lip and Palate; CP: Cleft palate only.

#### Materials

Speech materials comprise (1) counting from one to 10, (2) ten repetitions of all voiceless (or voiced where necessary) obstruents and sonorants in /aCa/, (3) sentences from the GOS.SP.ASS.’98,^21^ and (4) five minimal (pair) sets containing contrasting common substitutions for /s, ∫, t∫, t/ in a variety of vowel environments (for example, “seat, sheet, cheat, team, keep”). The full list is available in Cleland, Wrench, Lloyd and Sugden^22^ (see p27). For this study the ten repetitions of consonants were included for analysis. We selected /k, t, s, ʃ/ as high-pressure consonants representative of a variety of places and manner of articulation.

#### UTI Recording Set-Up

High-speed UTI data were acquired using a Micro machine controlled via a laptop running the Articulate Assistant Advanced software^TM^ version 2.17^21^ which recorded both audio and UTI. The echo return data were recorded at ∼100 fps over a field of view of either 144° or 162°. The field of view was selected with the probe positioned to allow the greatest view of the tongue, including both the hyoid and mandible shadows. The microconvex US probe was stabilized with a custom-made lightweight flexible plastic headset. For 6 children the probe was held in place by hand, either because the headset was too big, or the children requested not to use the headset. This may have affected the quality of the images, however DEI can be computed irrespective of translation and rotation. Data were collected in a quiet room at the Glasgow Dental Hospital before or after a routine appointment.

### Typically Developing Speakers: Ultrax Typically Developing

This dataset comprises 58 speakers (31 female and 27 male), aged 5–12 years (M = 9;7, SD = 2;1) from the east of Scotland. The dataset comprises two subsets of 30 and 28 children respectively, in subset one a wide selection of consonants and vowels were collected, in subset two data were designed to test whether children could learn non-English articulations and is therefore not of interest to this study. Speech materials in subset one were similar to the cleft dataset and were therefore appropriate for inclusion in this study. All children were monolingual English speakers and had typical receptive vocabulary as measured by the British Picture Vocabulary Scale^23^ and speech skills as measured by the Diagnostic Evaluation of Articulation and Phonology screen.^24^ None of the children made errors in production of the speech materials below. One child was excluded as he had a receptive vocabulary score outwith the normal range, leaving 29 participants’ data for analysis. Note, given the use of secondary data we were not able to match the groups for age and therefore the TD children were slightly older than the children with CL+/-P (*t*(58) = 4.53, *p *< .001). See table 1 for demographic details.

#### Materials: Typically Developing Children

Speech materials comprised: 1. The Diagnostic Evaluation of Articulation and Phonology Diagnostic Screen^24^ 2. Most consonants of English in a VCV environment with the Scottish English vowels /i,a,ʉ/ 3. The Scottish English vowels in a CVC environment, where the C was a bilabial 4. A range of English clusters in words and 5. A task designed to teach children non-English speech sounds using ultrasound biofeedback.^24^ For this study we selected the consonants /k, t, s, ʃ/ in an /aCa/ environment to compare to the cleft dataset. Note, there was only one repetition of these, rather than 10.

#### UTI Recording Set Up

The Ultrasound recording set up was similar to the cleft data set with the exception that the data were recorded with an Ultrasonix RP machine with a slightly higher frame rate of 121fps and a 112.5 degree field of view in the mid-sagittal plane. A metal headset was used to stabilise the probe. Comparisons between the lightweight plastic headset used for the cleft dataset and the metal version, show them to be similar.^25^

### Ultrasound Data Annotation

For both sets of data splines were fitted at the point of maximal lingual gesture/excursion of the consonant using AAA software.^26^ The frame with the maximal lingual gesture was identified manually by observing frame by frame within the consonant closure phase for stops and duration for fricatives. A spline was fitted to indicate the surface of the tongue using semi-automatic edge detection from the AAA software, which has recently been validated as a reliable method for tracking ultrasound tongue images.^13^ If small corrections were required, they were retraced manually using the AAA “snap-to-fit” function.

### Phonetic Transcriptions CP+/-L Group

For the children with CP+/-L ten repetitions of a range of consonants in an /aCa/ environment were phonetically transcribed by two clinicians who were experts in speech sound disorders using both the audio and ultrasound information. The full consonant set was : / p,m,f,w,θ,t,n,s,ɹ,l,ʃ,j,k,ŋ,tʃ /. The full method and results of these phonetic transcriptions is described in detail Cleland et al.^16^ In short this involved watching the ultrasound while transcribing and noting both a phonetic transcription and a classification of errors identifiable with the ultrasound image such as undershoot where the tongue approaches an articulatory target but does not reach it. Errors were classified as either developmental or non-developmental/cleft speech characteristics. Developmental/phonological errors and cleft speech characteristics were classified according to the processes listed in McLeod and Baker^27^ (p152–158 and p53 respectively) and tabulated (see table 2). Phonetic transcription results are presented for information, but not further analysed.

**Table 2.** Error Analysis for children with Cleft Palate +/- Cleft Lip based on transcription of single consonants in /aCa/.

	Developmental Errors						Cleft Speech Characteristics or Non-Developmental Errors													
							Backing to:													
Child	VF	Fric Simp	Stopping	Deaff	Labiodent /r/	Voicing	alveolar	Palatal	Velar	Uvular	Undershoot	Double Artic	Denasalised	Nasalised	Active Nasal Fric	Spirantised	Glottalised	Lateralised	Other	PCC
1		2							1		1	1	2							36
3	1		2				1	1	1					1	2					60
5		1													2		1		1	77
6	1	1					1		1	1	5					2			2	48
7					1															91
8		1		1	1	1		2	2		2								2	63
9											1								1	95
10		1								1					1					86
11		1	1					1	1		1					1			2	73
12	1								1		1								1	88
14	1							2	2			1		1			1		1	67
15		1			1							1					1		1	86
16														4			1			77
17	2	1		1			1	4	4		2									68
18	1																		1	95
19								2			12					1				0
20		1																	22	84
21																				100
22					1					1							1		3	91
23								2											2	91
24	1				1														11	84
25	1	1						1		1		3								53
26	1			1							1									93
27			2																	71
28								1								4	1	1		68
29										1							1	1		91
30														5			1			95
31	1							1						1		2		1	1	79
32									1					1						92
33	1	1				1					1			1						75
34					1			2	1		1	1								67
35	1			2				2	1		2									48
36	1						1												1	73
37	1				1			4											1	47
38	2	1	3	1	1															47
39					1			1							2					80

Table Note: VF = Velar Fronting, Fric Simp = Fricative Simplification, Deaff = Deaffrication, Labiodent /r/ = Labiodentalised /r/, Double Artic = Double Articulation. “Undershoot” = the Tongue Approaches a Target Obstruent Articulation but Does not Make Contact with the Palate. PCC = Percentage Consonants Correct.

### Calculation of DEI

DEI measures were automatically extracted for each of the consonants from every speaker and exported to a .csv file. AAA uses the DEI formula specified by Zharkova^18^ and described in the introduction. Higher DEI values indicate more dorsum excursion.

### Statistical Analyses

Statistical analyses were carried out using R Studio^28^ and data wrangling was performed using the ‘tidyverse’ package.^29^ A linear mixed effects model was run to test whether consonants at different places of articulation, /k, t, s, ʃ/, differ in DEI within TD children, and between TD children and children with CP+/-CL.^30^ The outcome variable was DEI, which was normally distributed upon visual inspection. The predictors were Diagnosis (TD was chosen as a baseline), Consonant (/ʃ/ was chosen as a baseline due to its intermediate postalveolar place of articulation), and the interaction between the two. The model^1^ also included random intercepts per speaker, accounting for the possibility that each speaker might have a systematically different DEI pattern. The model also included by-participant random slopes per consonant, to account for the possibility that each consonant might have a systematically different pattern within each child.

To test the effect of consonant on DEI within children with CP+/-CL, a linear mixed effects model was run on a subset containing the data of only children with CP+/-CL. It included DEI as an outcome variable and consonant as a predictor (/ʃ/ as baseline). It included the same random effects as the previous model: random intercepts per speaker and by-speaker random slopes per consonant.

## Results

Table 2 shows the error analysis for the children with CL+/-P. The typically developing children made no errors and are therefore not included in the table. Note both the wide range of errors and the presence of both developmental errors and cleft speech characteristics. Table 3 summaries the mean DEI per group and consonant. For the typically developing children, DEI values were within one standard deviation of the means presented by Zharkova^19^ as normative data, though note the DEI for /k/ was towards the lower end of this range. For the children with CP+/-CL, the DEI for /k/ was outwith the values given by Zharkova^19^ (0.36 compared to 0.51), however, all DEI values for this group were within one standard deviation of the typically developing children's values.

**Table 3.** Dorsum Excursion Index central tendency and spread values.

	Group	
Consonant	Typically Developing Mean (SD)	Cleft Lip and Palate Mean (SD)
∫	0.25 (0.08)	0.29 (0.11)
k	0.45 (0.14)	0.36 (0.13)
s	0.22 (0.07)	0.25 (0.10)
t	0.24 (0.05)	0.23 (0.09)

The effects of group and consonant and their interaction on DEI are summarized in Table 4.

**Table 4.** Results of the linear mixed effects model 1 focusing on the effect of consonant and diagnosis on DEI and model 2 focusing on the effect of consonant on children with cleft only.

Model 1						
Predictor	Estimate	Std. Error	df	t-value	p-value	
Intercept (TD, /ʃ/)	0.27	0.01	70.58	21.29	< 0.001	***
consonant (TD, /ʃ/ :/k/)	0.13	0.02	63.87	7.56	< 0.001	***
consonant (TD, /ʃ /:/s/)	−0.04	0.02	78.43	−2.33	0.02	*
consonant (TD, /ʃ /:/t/)	−0.04	0.01	82.92	−2.66	0.01	**
diagnosis (/ʃ/, TD:CLP)	0.02	0.02	70.58	1.39	0.17	
consonant (/ʃ/:/k/) : diagnosis (TD:CLP)	−0.09	0.02	63.87	−3.65	< 0.001	***
consonant (/ʃ/:/s/) : diagnosis (TD:CLP)	−0.01	0.02	78.43	−0.24	0.81	
consonant (/ʃ/:/t/) : diagnosis (TD:CLP)	−0.03	0.02	82.92	−1.45	0.15	
Model 2						
Predictor	Estimate	Std. Error	df	t-value	p-value	
Intercept (/ʃ/)	0.29	0.02	30.04	15.59	< 0.001	***
consonant (/ʃ/ :/k/)	0.07	0.02	29.74	3.17	0.004	***
consonant (/ʃ /:/s/)	−0.04	0.02	29.83	−1.83	0.08	
consonant (/ʃ /:/t/)	−0.06	0.02	28.37	−2.95	0.006	***

The results suggest that in TD children DEI is significantly higher for /k/ than for /ʃ/, and DEI is significantly lower for /s/ and /t/ than for /ʃ/. These outcomes are consistent with the prediction that more posterior places of articulation are associated with higher DEI.

**Figure 2.**
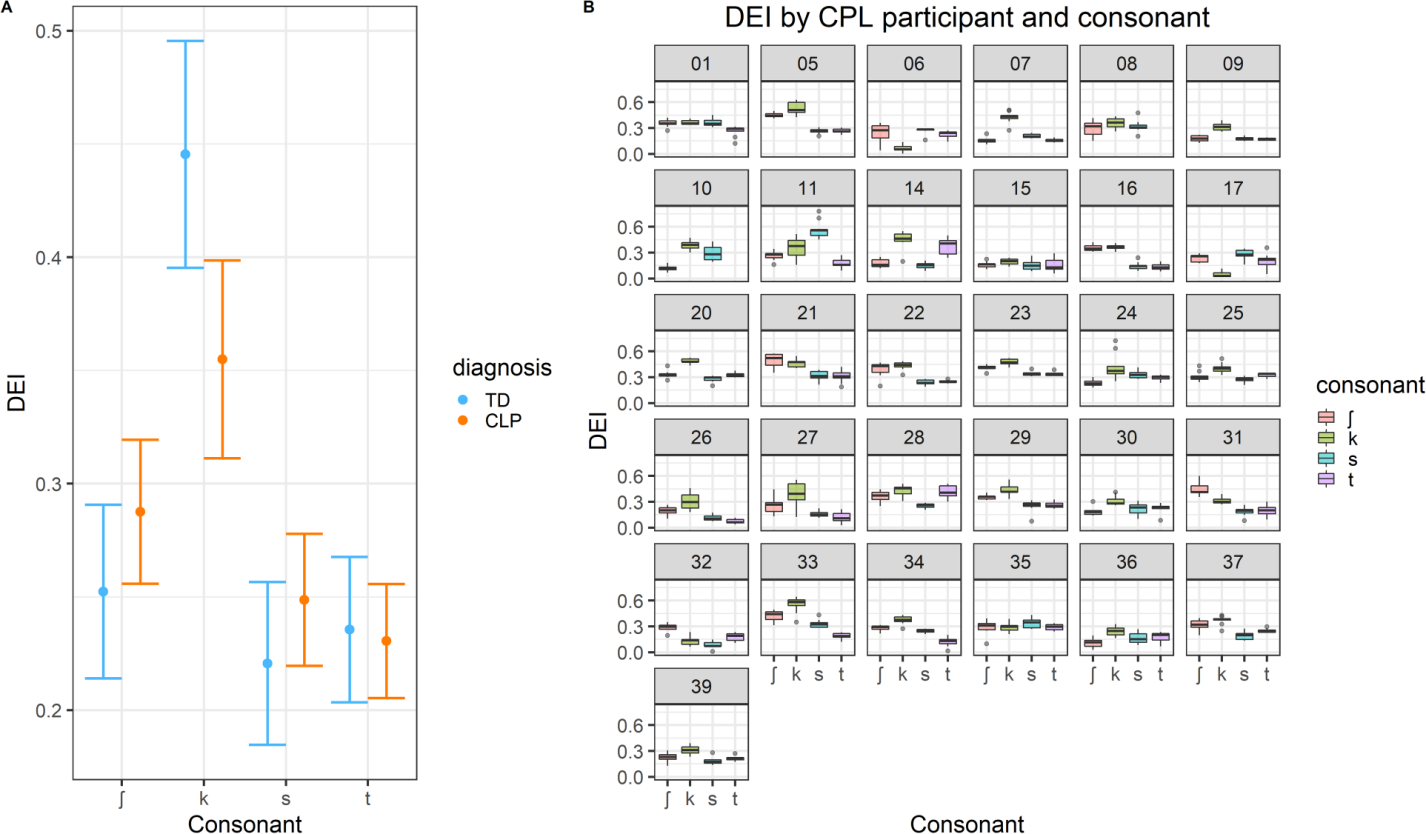
Left: model prediction of Dorsum Excursion Index per consonant for children with CP+/-CL and TD children. Right: Boxplot of the Dorsum Excursion Index per consonant for each child with CP+/-CL.

When comparing TD children and children with CP+/-CL, the only significant difference was between the position of /k/ relative to /ʃ/. The negative coefficient *β* = -0.09, suggests that the difference in DEI between /k/ and /ʃ/ is smaller in children with CP+/-CL than in TD children. This is also illustrated in the left hand panel of Figure 2. This result is not consistent with our hypothesis that children with CP+/-CL would have higher DEI than TD children due to increased raising of the tongue dorsum caused by a history of difficulty achieving velopharyngeal closure. In fact, it appears that the children in this study with CP+/-CL had a tendency for the developmental pattern of velar fronting. This is further explored below.

There were no significant differences between the two groups of speakers in the position of /ʃ/ on its own, and of /s/ and /t/ relative to /ʃ/. These results also do not provide support for the expectation of higher DEI in the CP+/-CL group.

The results of the second model shown in table 4 suggest that there was an effect of consonant on DEI in the CP+/-CL group only. /k/ had significantly higher DEI than /ʃ/, and /t/ had significantly lower DEI than /ʃ/. Both results are consistent with the prediction that the more posterior the place of articulation, the higher the DEI. There was no significant difference between /ʃ/ and /s/, which is perhaps a result of the higher variability in DEI per consonant within the CP+/-CL group than in the TD group, which is also illustrated in Figure 2.

To explore further the relatively lower DEI for /k/ in the CP+/-CL group, the right hand panel of Figure 2 shows DEI per consonant for each of the 31 children with CP+/-CL included in the analyses. While aggregate data and relative differences in DEI cannot serve as direct evidence for velar fronting, the figure suggests that many children had a DEI value for /k/ that was comparable to the DEI of other consonants. Table 2 shows that 15 speakers with CP+/-L showed some velar fronting (in some this was only fronting of the velar nasal which was not measured here, although for others there was clear fronting of /k/ to [t]), however almost all speakers showed retraction errors. This would suggest that the smaller difference between /k/ and /ʃ/ in the CP+/-CL group observed in Figure 2 cannot be entirely accounted for with velar fronting, but may be a mix of backing, fronting, and increased variability in the CP+/-CL group. Although the heterogeneity and size of the sample does not allow us to perform sub group analysis, it is worth highlighting that children with higher DEI values in anterior consonants did not necessarily have more retraction errors. For example, speaker 21 in the CP+/-CL group had consistently higher DEI values for the anterior consonants, yet these were all transcribed as correct.

## Discussion

This study used ultrasound tongue imaging and the dorsum excursion index to investigate whether there is evidence of increased raising of the tongue dorsum in children with repaired CP+/-CL compared to typically developing peers. We hypothesized that speakers with CP+/-CL would have a raised tongue dorsum compared to typical children across a variety of high-pressure lingual consonants, whether or not these consonants were in error due to compensation for a history of difficulties with velopharyngeal closure, also known as “lingual assistance”.^3^ Lingual assistance has previously been identified with both EPG^4^ and X-Ray.^5^ This difference in tongue posture may continue after surgery due to becoming habituated. Overall, our results did not support this hypothesis at a group level. Below we explore why this might be the case, as well as offering a critique of the DEI measure for distinguishing consonants within and across groups,^18^ and for measuring tongue dorsum raising. We conclude with a description of the limitations of our study which suggests that results be interpreted with caution.

### Increased Tongue Dorsum Raising in Children with CP+/-CL Compared to TD Children

We did not see across the board higher DEI values for this group of children with CP+/-CL. This was surprising given previous work by Gibbon^4^ that suggests that increased tongue-palate contact and retracted placement are due to overuse of the tongue dorsum. While the developmental error of velar fronting in some of the children with CL+/-P may have affected the findings for /k/, it was surprising that the other anterior consonants did not show higher DEI values, especially given patterns of backing in some children. We suggest that the key reason for not finding higher DEI values at group level is due to differences in the groups studied. Individual children likely present with increased DEI values. There were some key differences between our study and Gibbon's. Firstly, Gibbon^4^ is a summary of EPG studies collected over a long period of time from cleft palate clinics in the United Kingdom. Typically, the children attending these clinics were over the age of seven and had been selected for EPG therapy because they did not respond to previous treatment.^31^ This suggests they had more severe speech disorders than the children in our study who were recruited at routine appointments and may or may not have had overt speech disorders.^16^ In fact, the children in this study had percentage consonant correct values ranging from 0% correct to 100% correct.^16^ Secondly, EPG measures tongue palate contact, whereas ultrasound measures tongue-shape in the mid-sagittal view. It is possible that increased contact is a result of increased lateral bracing, which cannot be seen in mid-sagittal ultrasound. Studies are required which use both techniques to determine how comparable they are. However, both EPG and ultrasound can be used to identify retraction (for example, using qualitative measures as in^15^ and^16^), which is a typical feature of cleft palate speech, and indeed high levels of retraction were found in the dataset used for this study.^16^ However, some of the children in the current study also presented with the developmental process of velar fronting, which results in the converse: a fronted placement. This emphasizes the importance of future sub-group analyses. Lastly, both the Gibbon study^4^ and the Michi study^8^ report data from children that had their surgery and speech therapy several decades before the children in our study. In the UK there is evidence that speech outcomes have improved for children with CP+/-CL^32^ due to centralization of services and improved surgical timing and techniques. It is therefore plausible that increased tongue dorsum raising may now be more of an exception than a rule due to improved speech outcomes in this population.

### Using DEI to Distinguish Consonants within Speakers

In line with Zharkova,^19^ we predicted that consonants with more posterior places of articulation would have higher DEI values. Zharkova^19^ did not include /ʃ/ in her analyses but found that in an similar /a/ environment, /k/'s DEI was significantly different from all other consonants, /t/ was significantly different from /f/ and /p/, and /s/ was significantly different from /p/ and /ɹ/.

In TD children DEI followed the expected pattern of /k/>/ʃ/>/s/ and /ʃ/>/t/. It therefore appears that this is a reliable measure for distinguishing place of articulation in typical English speakers. Our reported values for typically developing children are in the range of those reported for six adults in Zharkova,^19^ although the value for /k/ was on the lower side. In children with CP+/-CL a similar pattern of DEI values was found despite these children presenting with speech errors. However, /k/ in this group had a relatively lower DEI value than the TD children suggesting possibly more fronted position- either to palatal or alveolar place of articulation. The values we present for typically developing children serve as potential norms for identifying errors or to measuring change over time. Given the values are similar to those for adults, we suggest that these serve as useful norms for speakers over the age of five. Norms for younger children, who are still in the process of developing intelligible speech would be useful.

### Limitations and Future Directions

Our study is limited by the use of only one ultrasound metric, which although designed for use with CP+/-CL, had prior to this study not been trialed with this population. DEI is a scaler metric.^10^ This means it yields a ratio value with no units. This is useful because it can be computed irrespective of translation or rotation of the image, both within and across speakers. However it also means that DEI measures the degree to which the tongue is in a bunched configuration, rather than necessarily how far back in the vocal tract the articulation is made. For typically developing English speaking children, these concepts are related because velar consonants (measured here) are produced with the back of the tongue resulting in a more bunched configuration, i.e., more dorsum excursion (see above) than alveolar consonants. However, for speakers with CL+/-P it would also be useful to have a measure of how far back in the vocal tract an articulation is made. For example, figure 1 shows a similar tongue shape for [q] and [k]. The small difference in DEI value is driven by the fact that the midpoint of line n falls slightly nearer to the maximum tongue height, not necessarily by the fact that the tongue shape for [q] is further back in the vocal tract. Further work is needed comparing DEI with other metrics. For example, if the probe is stabilized relative to head movement then it would be possible to compare the location of the highest point of the tongue between different articulations.

Our study is also limited by its use of pre-existing datasets and therefore the types of speech materials, age range, and number of repetitions differed between groups. As a result, the analyses include speakers with a wide age range. The children with CP+/-L therefore had some developmental errors, such as velar fronting, which were not present in the control group. This likely affected the results for /k/, where the DEI was lower than expected in the CP+/-L group. For both groups of children, we used non-lexical data e.g., /aka/. Although these data were relatively consistent and straightforward to analyze, it is possible that we may have found more differences between groups, or more evidence of a raised tongue body, if we had used materials which are more articulatorily complex, especially with regard to the velopharyngeal mechanism. Likewise, the number of repetitions differed between groups, with one repetition in the TD group, therefore limiting the possibility of comparing stability of DEI across multiple repetitions in groups. Similarly, we chose to only analyze the maximum point of constriction, rather than multiple time points. However, we would suggest that if tongue dorsum raising occurs, then this would be represented at this articulatory landmark. Similarly, the consonants chosen for analysis were high-pressure fricatives and plosives, which again ought to be vulnerable to increased tongue dorsum raising due to lingual assistance.^4^ A future possible angle would be to compare DEI in nasal consonants and/or vowels to high pressure consonants to determine whether there are differences in tongue dorsum raising. Moreover, our study is limited by analysis of a single language. It would be particularly useful to collective normative DEI values for posterior consonants such as uvulars and pharyngeals which occur in languages other than English. Lastly, we suggest that use of a larger dataset of children with CP+/-CL would enable analyses which can determine whether some subgroups of children, perhaps those with noticeable hypernasality or nasal emission, show increased DEI values.

## Conclusion

This study sought to determine whether children with CP+/-CL show increased raising of the tongue dorsum compared to typically developing children. Our preliminary results do not support the hypothesis that this pattern is ubiquitous in this population, as measured using the dorsum excursion index. However, our results must be interpreted with caution because the speakers in this study had a wide range of speech sound disorders, ranging from typical/resolved speech to severely unintelligible speech, as well as a wide age range. We suggest that some speakers with CP+/-CL may show this pattern in their speech, but it is likely to be the minority. We reported values for the Dorsum Excursion Index^19^ for a range of consonants in speakers with CP+/-CL and typically developing children. In general, these were within the range previously reported for typical adult speakers.^19^ These values therefore have the potential to be used as norms. This would be a particularly useful metric for quantifying intervention outcomes in children undergoing ultrasound visual biofeedback therapy for errors involving fronting or backing errors.^11^ For determining whether an individual speaker with CP+/-L presents with increased raising of the tongue dorsum, we suggest combing the DEI measure with phonetic transcription to exclude any developmental errors.

## Notes

DEI ∼ consonant*diagnosis + (1 + consonant |speaker)

## References

[1] Sell D . Issues in perceptual speech analysis in cleft palate and related disorders: A review. Int J Lang Commun Disord. 2005;40(2):103-121. doi:10.1080/13682820400016522 16101269

[2] Nagarajan R Savitha VH Subramaniyan B . Communication disorders in individuals with cleft lip and palate: An overview. ijplasurg. 2009;42(S01):137-143. doi:10.1055/s-0039-1699387 PMC2825064 19884669

[3] Trost JE . Articulatory additions to the classical description of the speech of persons with cleft palate. Cleft Palate J. 1981;18(3):193-203. 6941865

[4] Gibbon FE . Abnormal patterns of tongue-palate contact in the speech of individuals with cleft palate. Clin Linguist Phon. 2004;18(4-5):285-311. doi:10.1080/02699200410001663362 15259573

[5] Yamashita Y Michi K-I . Misarticulation caused by abnormal lingual-palatal contact in patients with cleft palate with adequate velopharyngeal function. Cleft Palate Craniofac J. 1991;28(4):360-368. doi:10.1597/1545-1569_1991_028_0360_mcbalp_2.3.co_2 1742304

[6] Gibbon F Smeaton-Ewins P Crampin L . Tongue-Palate contact during selected vowels in children with cleft palate. Folia Phoniatr Logop. 2005;57(4):181-192. doi:10.1159/000085186 16037694

[7] He F Wang X Yin H Zhang H Yang G He L . Acoustic analysis and detection of pharyngeal fricative in cleft palate speech using correlation of signals in independent frequency bands and octave spectrum prominent peak. Biomed Eng Online. May 27 2020;19(1):36. doi:10.1186/s12938-020-00782-3 32460765 10.1186/s12938-020-00782-3 PMC7251748

[8] Michi KY Y. Imai S Ohno K . Results of treatment of speech disorders in cleft palate patients: patients obtaining adeqate velopharygenal function. In: Pfeifer G , ed. Craniofacial anomalies and clefts of the lip alveolus and palate. Thieme; 1990:419-423.

[9] Cleland J Preston J . Biofeedback interventions. In: Williams AL McLeod S McCauley R , eds. Interventions for speech sound disorders in children. Vol chap 20. Pearson; 2021:573-599.

[10] Cleland J . Ultrasound tongue imaging. Manual of clinical phonetics. Routledge; 2021:399-416.

[11] Sugden E Lloyd S Lam J Cleland J . Systematic review of ultrasound visual biofeedback in intervention for speech sound disorders. Int J Lang Commun Disord. 2019;54(5):705-728. doi:10.1111/1460-6984.12478 31179581

[12] Lawson E Stuart-Smith J Scobbie J, M. Nakai S. Seeing Speech: An articulatory web resource for the study of Phonetics. University of Glasgow Accessed 19 January, 2023. https://seeingspeech.ac.uk

[13] Roon KD Chen W-R Iwasaki R , et al. Comparison of auto-contouring and hand-contouring of ultrasound images of the tongue surface. Clin Linguist Phon. 2022;36(12):1112. doi:10.1080/02699206.2021.1998633 34974782 PMC9250540

[14] Wrench A Balch-Tomes J . Beyond the edge: Markerless pose estimation of speech articulators from ultrasound and camera images using DeepLabCut. Sensors. 2022;22(3):1133. 35161879 10.3390/s22031133 PMC8838804

[15] Bressmann T Radovanovic B Kulkarni GV Klaiman P Fisher D . An ultrasonographic investigation of cleft-type compensatory articulations of voiceless velar stops. Clin Linguist Phon. 2011;25(11-12):1028-1033. doi:10.3109/02699206.2011.599472 21787146

[16] Cleland J Lloyd S Campbell L , et al. The impact of real-time articulatory information on phonetic transcription: Ultrasound-aided transcription in cleft lip and palate speech. Folia Phoniatr Logop. 2020;72(2):120-130. doi:10.1159/000499753 31129664

[17] Roxburgh Z Cleland J Scobbie JM Wood SE . Quantifying changes in ultrasound tongue-shape pre- and post-intervention in speakers with submucous cleft palate: An illustrative case study. Clin Linguist Phon. 2021;36(2-3):146-164. doi:10.1080/02699206.2021.1973566 34496688

[18] Zharkova N . Using ultrasound to quantify tongue shape and movement characteristics. Cleft Palate Craniofac J. 2013;50(1):76-81. doi:10.1597/11-196 22117937

[19] Zharkova N . A normative-speaker validation study of two indices developed to quantify tongue dorsum activity from midsagittal tongue shapes. Clin Linguist Phon. 2013;27(6-7):484-496. doi:10.3109/02699206.2013.778903 23651147

[20] Eshky A Ribeiro MS Cleland J , et al. UltraSuite: a repository of ultrasound and acoustic data from child speech therapy sessions. presented at: Interspeech 2018; 2018; https://strathprints.strath.ac.uk/64824/

[21] Sell D Harding A Grunwell P . GOS.SP.ASS.'98: An assessment for speech disorders associated with cleft palate and/or velopharyngeal dysfunction (revised). Int J Lang Commun Disord. 1999;34(1):17-33. doi:10.1080/136828299247595 10505144

[22] Cleland J Wrench A Lloyd S Sugden E . ULTRAX2020: ultrasound technology for optimising the treatment of speech disorders: clinicians’ resource manual. University of Strathclyde; 2018:87.

[23] Dunn LM Dunn DM Styles B Sewell J . British picture vocabulary scales III. NFER-Nelson; 2009.

[24] Dodd B Zhu H Crosbie S Holm A Ozanne A . Diagnostic evaluation of articulation and phonology (DEAP). Psychology Corporation; 2002.

[25] Pucher M Klingler N Luttenberger J Spreafico L . Accuracy, recording interference, and articulatory quality of headsets for ultrasound recordings. Speech Commun. 2020;123:83-97. doi:10.1016/j.specom.2020.07.001

[26] *Articulate Assistant Advanced Ultrasound Module user manual, revision 2* *.16*. Articulate Instruments; 2014.

[27] McLeod S Baker E. *Children's speech: An evidence-based approach to assessment and intervention* . 2017.

[28] Team R. *RStudio: Integrated Development for R* *.* *RStudio, PBC* . 2020.

[29] Wickham H Averick M Bryan J , et al. Welcome to the tidyverse. Journal of Open Source Software. 2019;4(43):1686. doi:10.21105/joss.01686

[30] Bates D Mächler M Bolker B Walker S . Fitting linear mixed-effects models using lme4. J Stat Softw. 10/07 2015;67(1):1-48. doi:10.18637/jss.v067.i01 doi:10.18637/jss.v067.i01

[31] Lee ASY Law J Gibbon FE . Electropalatography for articulation disorders associated with cleft palate. Cochrane Database Syst Rev. 2009;3:1-20. doi:10.1002/14651858.CD006854.pub2 PMC7390345 19588407

[32] Sell D Mildinhall S Albery L Wills AK Sandy JR Ness AR . The cleft care UK study. Part 4: Perceptual speech outcomes. Orthod Craniofac Res. 2015;18(S2):36-46. doi:10.1111/ocr.12112 26567854 PMC4670716

